# Unmasking the biological function and regulatory mechanism of NOC2L: a novel inhibitor of histone acetyltransferase

**DOI:** 10.1186/s12967-023-03877-2

**Published:** 2023-01-17

**Authors:** Siyi Lu, Zhaoyu Chen, Zhenzhen Liu, Zhentao Liu

**Affiliations:** 1 Department of General Surgery, Peking University Third Hospital, Beijing, 100191 China; 2 Department of Basic Medical Sciences, Peking University Health Science Center, Beijing, 100191 China; 3 Department of Thoracic Surgery, Beijing Jishuitan Hospital, Beijing, 100035 China; 4 Department of Medical Oncology and Radiation Sickness, Peking University Third Hospital, Beijing, 100191 China

**Keywords:** NOC2 like nucleolar associated transcriptional repressor (NOC2L), Inhibitor of histone acetyltransferase (INHAT), P53, Transcriptional regulation, Embryonic development, Carcinogenesis

## Abstract

NOC2 like nucleolar associated transcriptional repressor (NOC2L) was recently identified as a novel inhibitor of histone acetyltransferase (INHAT). NOC2L is found to have two INHAT function domains and regulates histone acetylation in a histone deacetylases (HDAC) independent manner, which is distinct from other INHATs. In this review, we summarize the biological function of NOC2L in histone acetylation regulation, P53-mediated transcription, ribosome RNA processing, certain development events and carcinogenesis. We propose that NOC2L may be explored as a potential biomarker and a therapeutic target in clinical practice.

**Supplementary Information:**

The online version contains supplementary material available at 10.1186/s12967-023-03877-2.

## Background

In the nucleus of cell, the acetylation and de-acetylation of histone is in a dynamic balance, which precisely regulates gene transcription and expression [1, 2]. Histone acetylation, which majorly happens in the lysine residues of histone tails, belongs to one type of various histone modification models. Other common types of histone modifications include phosphorylation and methylation [2, 3]. Studies show that the transcriptional activity is highly associated with the acetylation level of histone. Nucleosomes in transcription active regions tend to exhibit a high degree of acetylation, while those in transcription inactive regions are more likely to be acetylated in a low level [3]. The key molecules involved in this dynamic process are histone acetyltransferase (HAT) and histone deacetylases (HDAC), which add acetyl group to histone and remove it from histone respectively [4, 5]. The histone acetylation modification has a profound impact on cell division, proliferation, differentiation, apoptosis, migration, and other important processes [2, 6]. Therefore, it is important to investigate the regulation of histone acetylation and identify key regulatory molecules.

NOC2 like nucleolar associated transcriptional repressor (NOC2L) is an inhibitor of histone acetylation [7–11]. Specifically, NOC2L is identified as an inhibitor of histone acetyltransferase (INHAT), which inhibits HAT by binding to histone tails and inhibiting the interaction between HAT and histone [7]. More importantly, it is a novel inhibitor which functions independently of HDAC, making it unique from other INHATs [7].

As a novel HDAC-independent INHAT, NOC2L provides a novel insight into the investigation of histone acetylation. In order to delineate the biological function of NOC2L, further studies were conducted to investigate NOC2L-targeted proteins and the underlying regulation mechanisms. NOC2L has been demonstrated to target TP53 [7, 8, 10, 11], TP63 [11], Aurora B [10], Mouse double minute 2 homolog (MDM2) [8], retinoblastoma protein (RB) [9], Enhancer of zeste homolog 2 (EZH2) [12]. In this review, we summarize the biological function of NOC2L and the underlying mechanisms of how NOC2L interacts with its key targets.

## *NOC2L* gene cloning and identification

As a protein coding gene, *NOC2L* (Gene Bank ACCESSION NM: NM_015658.4, Gene ID: 26155) is also known as NIR, NET7, NET15 and PPP1R112. *NIR* is commonly used as the synonyms of *NOC2L*. *NOC2L* was first identified by Hublitz et al. in an attempt to find novel INHAT by conducting low-stringency iterative analyses of the Basic Local Alignment Search Tool (BLAST) to screen databases for proteins containing putative INHAT regions. A cDNA coding for an unknown protein was identified and its human full-length sequence was obtained by PCR from a prostate cDNA library [7].

*NOC2L* gene is located on chromosome at 1p36.33 and contains 19 exons with an open reading frame including 2757 nucleotides, according to the BLAST analysis.

### Structure and localization of NOC2L protein

**Fig. 1:**
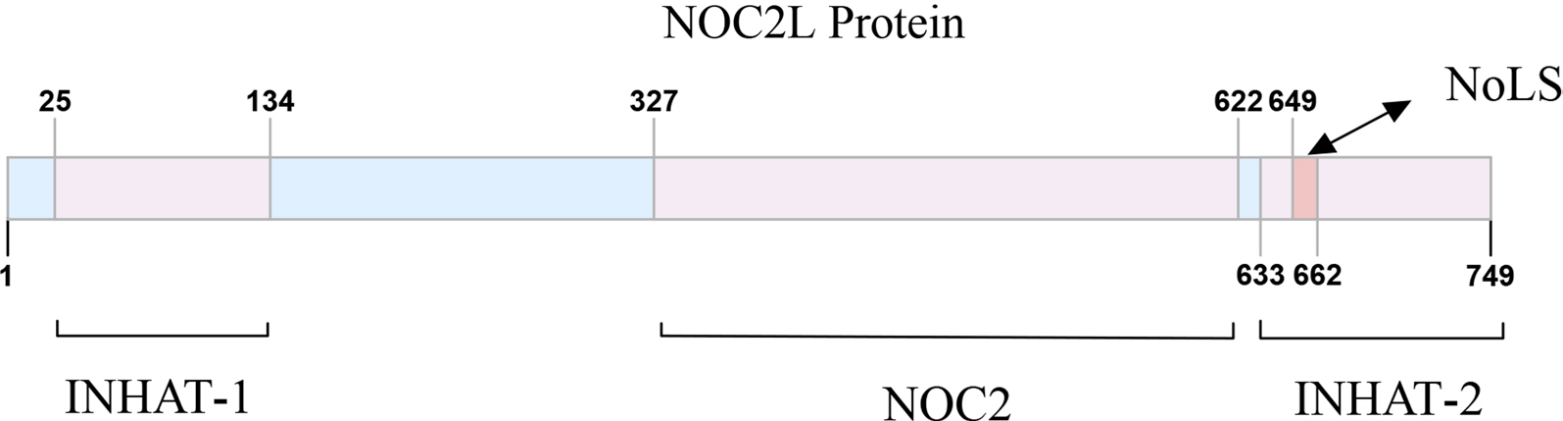
Structure of NOC2L Protein

NOC2L protein is 84,919 Da and contains 749 amino acids in human, and its orthologs range from worms to mammals (NCBI Reference Sequence: NP_056473.3). NOC2L is located at the nucleolus primarily, as is indicated in the Human Protein Atlas (HPA) database [13]. However, study also shows that the presence of a small amount of NOC2L can also be detected in the nucleoplasm, where various transcriptional factors and their corresponding regulators reside [7, 11]. The structure of NOC2L protein, as shown in Fig. 1, includes the two INHAT function domains (Amino acids 25-134, Amino acids 633-749), the conserved NOC2 domain (Amino acids 327-622) and the nucleolar localization sequence (NoLS) domain (amino acids 645-666) [14]. In this case, NOC2L is divergent from other INHATs due to its unique composition of two functional domains, which are located at its N terminus and C terminus respectively. Using Nucleolar localization sequence detector (NoD) [15], the NoLS of NOC2L is predicted to be FPEIKRRKMADRKDEDRKQFKD in NOC2L protein.

### Expression profile of *NOC2L* in normal tissue

**Fig. 2:**
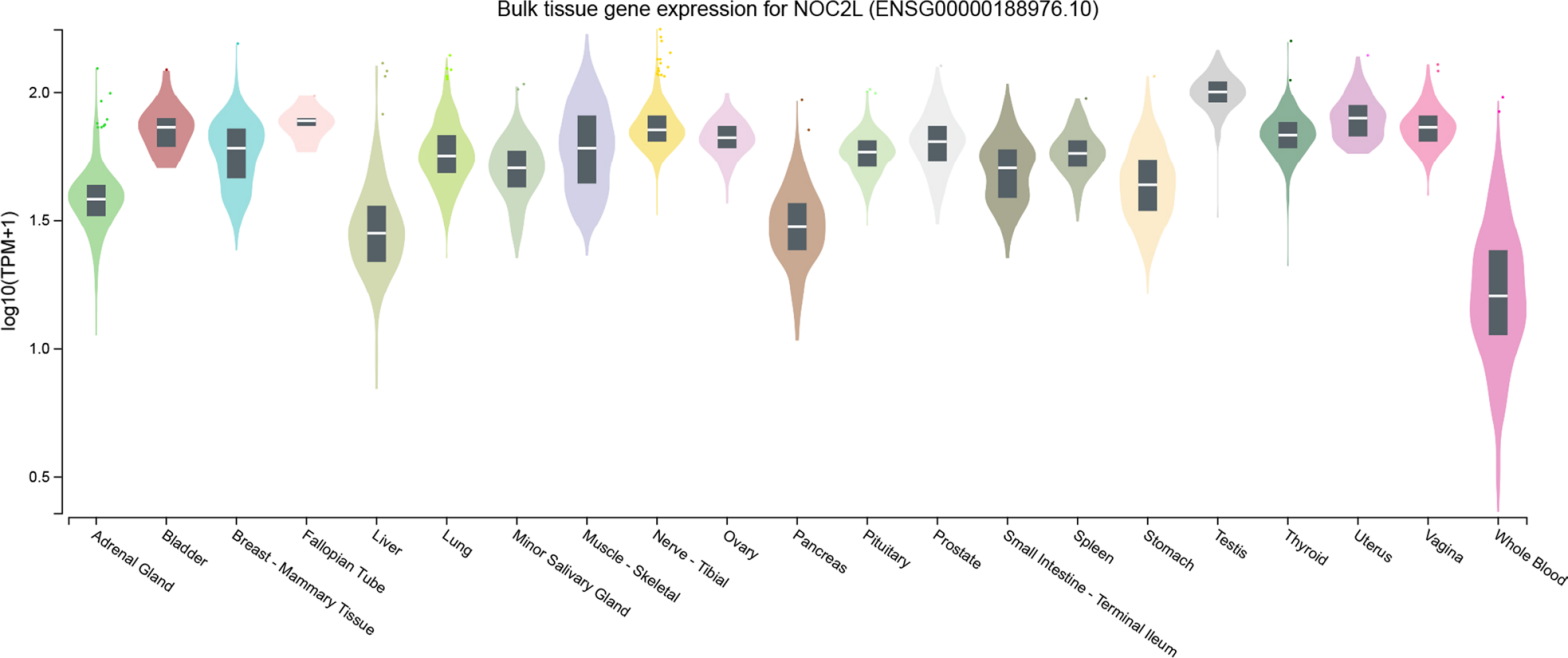
Bulk Tissue Gene Expression for *NOC2L*

The expression profile of *NOC2L* is wide and ubiquitous, as indicated in the Genotype-Tissue Expression (GTEx) database (Fig. 2) [16]. *NOC2L* is expressed universally during the entire process of embryonic development, and widely discovered in all adult tissues [7].

### Biological function of NOC2L

The basic function of NOC2L is to inhibit histone acetylation by inhibiting HAT activity. Almost all the underlying mechanisms of the biological events affected by NOC2L is based on its basic function as an INHAT.

NOC2L was identified as an INHAT by screening protein database for INHAT-domain containing protein [7]. As indicated before, NOC2L contains two putative INHAT domains, and both have been verified to physically interact with core histones and nucleosomes [7]. Furthermore, there is a direct interaction between NOC2L and the N-terminal histone H3 tail (Amino acids 1–30). However, when the H3 tail is already acetylated, no association of NOC2L with histone can be observed [7, 17]. Both INHAT domains in NOC2L can significantly reduce acetylation by HAT p300 of all the core histones, and acetylation by another HAT p/CAF of histone H3 and H4 [7, 18, 19].

**Fig. 3:**
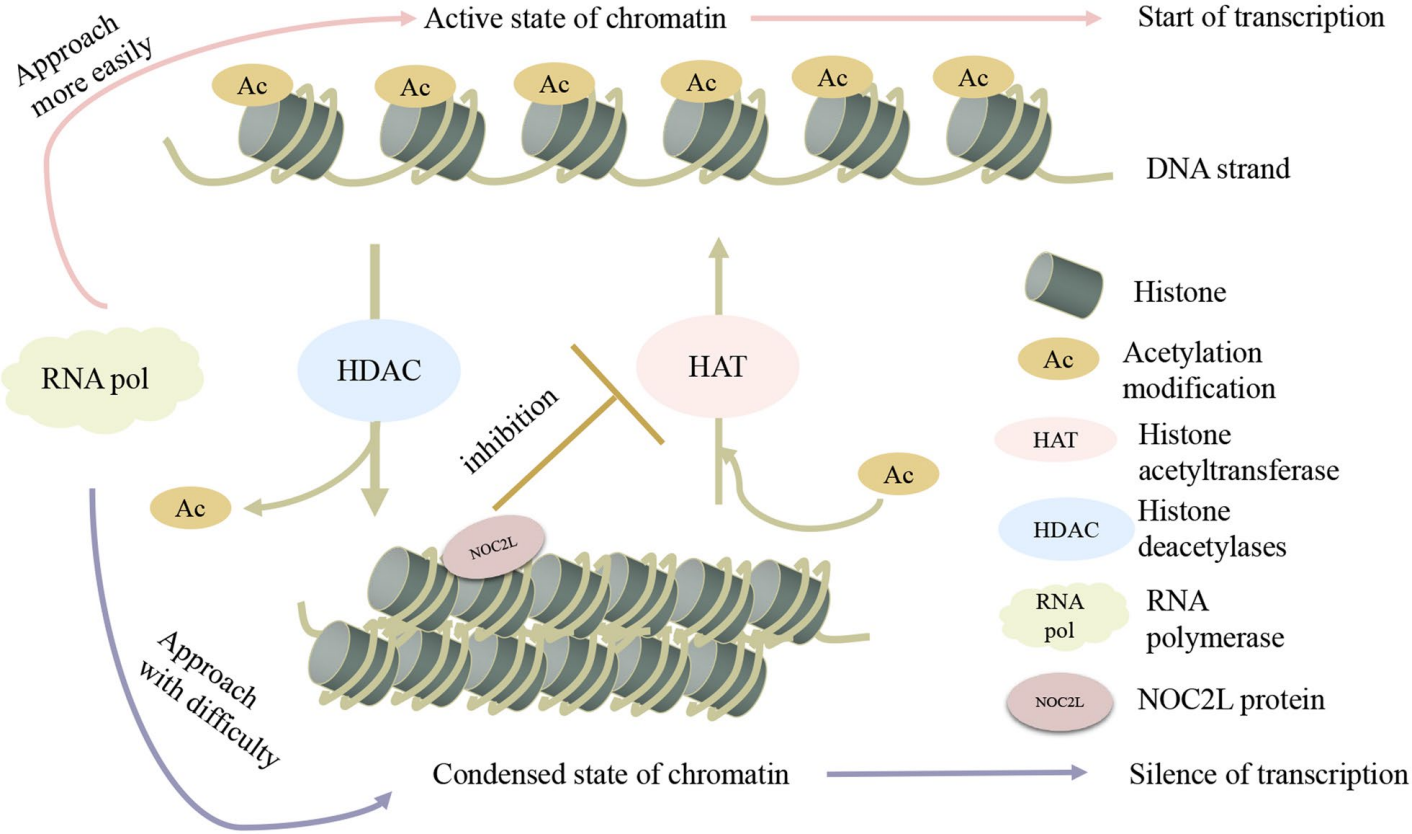
INHAT Function of NOC2L. In the compacted state of chromatin, histones form a spatial barrier for the RNA-polymerase to access to the DNA, which wind around these histones. When the lysine residue of histone is acetylated by HAT, the positive charge of lysine is neutralized, thus weakening the interactions between DNA and histones, allowing more space and making it more easily for RNA-polymerase to approach to the exposed DNA. When HDAC removes acetylation groups from histone, chromatin returns to the compacted state, forming a spatial barrier and making it difficult for RNA-polymerase to approach. When NOC2L occupies histone, it inhibits HAT activity to transfer acetylation group to the NOC2L-occupied histone. The acetylation level of histone is reduced, and the chromatin is in a condensed state where transcription is inactivated

According to these observations, NOC2L can bind to histone tails and inhibit histone acetylation [7]. The underlying molecular basis of NOC2L function can be speculated as follows (Fig. 3). Both HAT and NOC2L can bind to histone, which can be considered as the substrate of HAT [20]. The binding of NOC2L to histone inhibits HAT from accessing to its substrate, and HAT cannot deliver acetylation group to its substrate any more, similar to the mechanism of other INHATs [17, 21–23].Consequently, the acetylation of histone is reduced [17, 21–23]. In conclusion, NOC2L may serve as a histone competitive binding inhibitor of HAT [17, 20–23] and more knowledge on the molecular basis of INHAT function remains to be further elucidated.

As indicated above, NOC2L is identified as a novel HDAC-independent inhibitor of HAT, which makes it unique from other previously identified INHATs [20]. Other INHATs, such as pp32, are dependent of HDAC, because they recruit HDAC to histone to remove acetylation groups [21]. These INHATs inhibit histone acetylation by both inhibiting HAT and recruiting HDAC. In the presence of HDAC, the effect of these INHATs in inhibiting acetylation is more significant [21]. In contrast, experimental evidence from the work conducted by Hublitz et al. [7] indicates that immunoprecipitated NOC2L does not physically associate with HDAC, and the induction of HDAC inhibitor does not influence NOC2L-mediated inhibition of transcription, which suggests that NOC2L does not rely on HDAC to facilitate it in inhibiting histone acetylation [7].

In conclusion, NOC2L is a novel and potent INHAT which functions in a HDAC-independent manner [7–11]. Despite the abundant knowledge of the regulation of histone acetylation, the HDAC-independent histone acetylation regulation remains to be elucidated [22, 23]. Therefore, the further investigation of NOC2L is crucial for the deeper understanding of histone acetylation regulation.

### A key target of NOC2L: P53 and P53-associated molecules

#### NOC2L modulates P53 function through direct interaction with P53

After identifying NOC2L as a novel HDAC-independent INHAT, subsequent work was conducted to enhance the understanding of the biological function of NOC2L. Scientists first sought to analyze NOC2L-associated proteins by mass spectrometry [7]. Among the various proteins identified by this approach, P53 caught their attention.

P53 has been described as the most studied gene in the human genome and enjoys the reputation as ‘guardian of the genome’ [24], which is involved in multiple important biological processes including apoptosis, cell growth and senescence [25]. It is a tumor suppressor which functions primarily as a transcription factor. When various stress signals trigger its activation, it will bind to the promoter region of its target gene and recruit other transcriptional cofactors [24]. The transcription of downstream genes can lead to apoptosis and cell-cycle arrest [26].

The in vitro and in vivo association of NOC2L and P53 were verified by GST-pulldown and immunoprecipitation assay respectively [7]. According to bioinformatic analysis, the central part (Amino acids 147-609) of NOC2L interacts with P53 [7]. The DNA-binding domain (DBD, amino acids 102-292) and the C-terminal tetramerization domain (Amino acids 293-359) of P53 interacts with NOC2L [7]. In summary, NOC2L was identified as a P53-interacting partner [7, 8, 10, 11]. It is worth mentioning that NOC2L is the first INHAT identified to associate with P53, leading the investigation of NOC2L to be more meaningful and promising [7, 8, 10, 11].

**Fig. 4:**
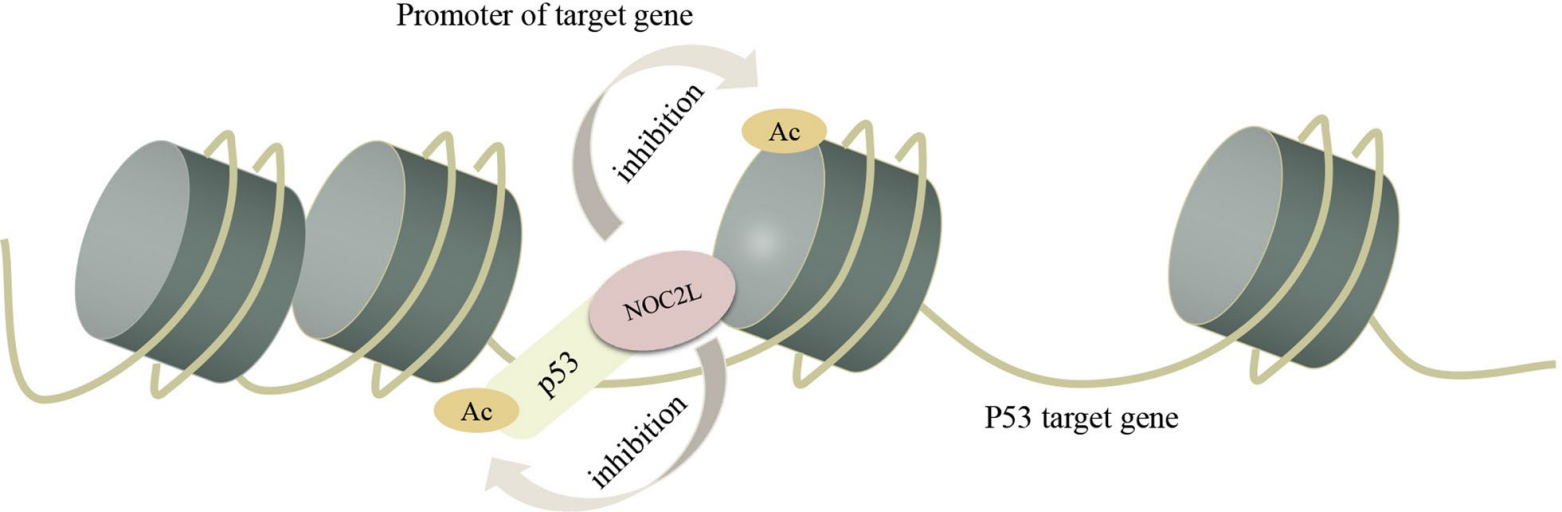
NOC2L Interacts with P53 on P53-targeted Gene. Upon recruitment by P53, NOC2L regulates the acetylation status in the vicinity of P53-occupying sites. NOC2L inhibits the acetylation of H3/H4 at p53 target promoters. Due to the inhibition of histone acetylation, the transcription of P53 target gene is inhibited

NOC2L is recruited by P53 to inhibit histone acetylation in P53-targeted gene [7, 8, 10, 11]. After NOC2L overexpression, the level of histone acetylation in the promoter region decreases [7], which leads to the inhibition of p53-mediated transcription (Fig. 4).

Besides blocking the histone acetylation, NOC2L also changes the acetylation level of P53 [7, 11], but how this affects transcription was not investigated further [7]. However, recent studies suggest that the effect of acetylation on P53 function is quite controversial and depends on the context [27]. Based on the fact that NOC2L has been demonstrated to inhibit the acetylation of P53 [7, 8], RB [9], and MDM2 [8], we can speculate that NOC2L may not act merely as an inhibitor of histone acetylation but possess a wider ability to inhibit the acetylation of other NOC2L-associated proteins [7–9]. Indeed, this speculation still needs further investigation to be confirmed.

Hublitz et. al further investigated the consequence of *NOC2L* expression on the P53 target genes. NOC2L can inhibit the transcription and expression of several P53-mediated genes, such as *P21*, *PIG3* and *NOXA*. And the inhibition ability of NOC2L is dependent on P53 [28]. NOC2L can easily inhibit the transcription and expression of *P21* without the treatment of DNA-damaging agent doxorubicin (DOX) because *P21* promoter has a higher P53 occupation level than other P53 target genes, such as *NOXA* and *PIG3 *[7]. This P53-dependent characteristic confirms that NOC2L is recruited by P53 to regulate the histone acetylation of P53 target genes. The repression of P53 target genes have a significant impact on cell fate. Upon *NOC2L* knockdown, increased apoptosis was observed in P53-containing cells but not in P53-deficient cells [7, 8, 10, 11]. This demonstrates that NOC2L inhibits P53-dependent apoptosis.

Before the identification of NOC2L, other INHATs have not been demonstrated to physically interact or functionally associate with P53 [7, 17, 21], suggesting that NOC2L is the first INHAT known to associate with P53. NOC2L can inhibit the histone acetylation in the promoter of P53 target genes. Upon NOC2L association, P53-activated gene expression is inhibited, and P53-dependent apoptosis is repressed. NOC2L can be regarded as a co-transcription factor of P53 target genes. Unlike other co-transcription factors that bind to P53, NOC2L is a negative regulator of P53 activity while most co-transcription factors are positive regulator elements of P53 [29]. Considering the significant role of P53 in cellular growth, the investigation of the interaction between NOC2L and P53 may shed light on the deeper understanding of the P53-mediated transcriptional mechanism.

#### NOC2L modulates P53 function through interaction with other proteins

P53 is regulated by multiple proteins, which can influence the function of P53 [24–26]. Since NOC2L can regulate P53 [7], there is possibility that NOC2L may affect P53 function by both directly interact with P53 and interact with other P53-associated proteins to indirectly impact P53 through their interactions [7, 8, 10].

#### NOC2L interacts with Aurora B kinase to suppress P53 activity

During the investigation of NOC2L’s biological function, Aurora kinase B (*AURKB*), a highly expressed serine and threonine protein kinase [30],was also identified to interact with NOC2L [10]. As a member of Aurora kinases, Aurora B plays an indispensable part in maintaining the integrity of a cell during cell division [31]. Aurora B can regulate cell cycle by directing chromosomal and cytoskeletal movements during mitosis [32, 33].

Previous studies indicated that P53 can be upregulated by the treatment of an Aurora B kinase inhibitor called AZD1152-HQPA in acute myelogenous leukemia cells, suggesting a functional connection between Aurora B and P53 [34]. Aurora B may regulate P53 function through phosphorylation at P53 amino terminus, which is the most studied post-translational modifications in P53 [35]. Phosphorylation at most these sites can improve the stability and function of P53, while certain phosphorylation sites on P53 display a negative effect to inactivate P53 [36, 37]. Therefore, we may speculate that Aurora B can inactivate P53 through the negative regulatory sites of phosphorylation on P53.

Wu et. al demonstrated that Aurora B, NOC2L and P53 form a complex in which NOC2L bridges Aurora B to phosphorylate P53 mainly within the DBD, leading to the repression of P53 function [10].NOC2L binds to Aurora B in its kinase domain, and the binding site in NOC2L is its N (1–250 amino acids) and C terminus (500–749 amino acids) [10]. Since both Aurora B [10] and P53 [7] have been identified to partner with NOC2L, it can be speculated that NOC2L may affect P53 function through regulating the kinase activity of Aurora B. Based on this hypothesis, further investigation was conducted to verify the possibility that Aurora B kinase may serve as a negative regulator of P53 upon phosphorylation modification [10]. In accordance with the speculation, Aurora B was demonstrated to phosphorylate P53 on Ser183, Ser269 and Thr284 [10]. Among the three phosphorylation sites, Ser269 and Thr284 showed significant ability to suppress P53 activity by affecting its transcriptional activity and inducing P53-dependent apoptosis [10]. It is worth noting that all these phosphorylation sites are located in the DBD of P53, which may further our understanding of the phosphorylation modification in P53 DBD [10].

**Fig. 5:**
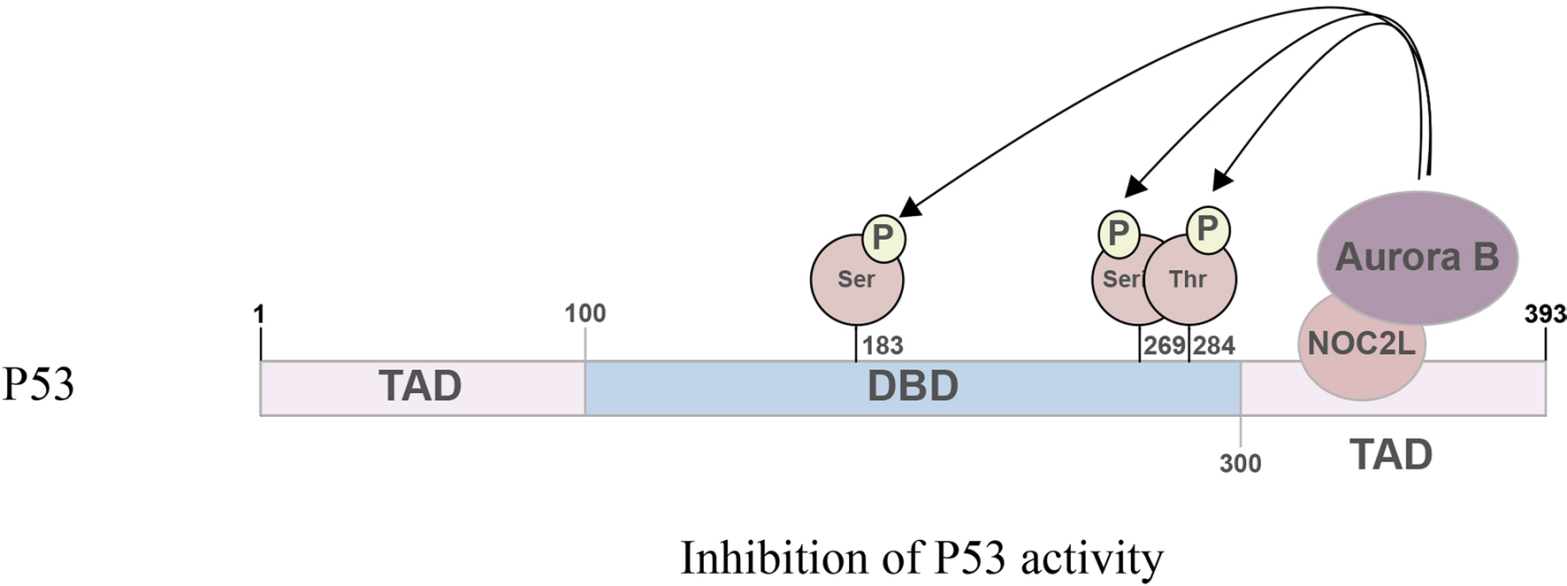
NOC2L, Aurora B and P53 Form a Complex. NOC2L binds to P53, and recruits Aurora B to phosphorylate P53 on Ser183, Ser269 and Thr284 sites. After phosphorylation, P53 activity is inhibited

Based on the findings, a model was proposed that the P53-NOC2L-Aurora B complex represses P53 function through phosphorylation by Aurora B under physical conditions, as indicated in Fig. 5. NOC2L binds to P53, and recruits Aurora B to phosphorylate P53 on Ser183, Ser269 and Thr284. The phosphorylation of Aurora B on P53 will inactivate P53 [10]. This discovery proposes a novel perspective on the NOC2L-regulated P53 repression and provides an additional pathway of P53 regulation.

#### NOC2L interacts with the P53-inhibiting ubiquitin ligase MDM2

MDM2 is another important binding partner of NOC2L [8]. MDM2 is an E3 ubiquitin ligase regarded as the most important P53 negative regulator. P53 can be rapidly degraded and exported from the nucleus upon association with MDM2 [38–40]. It is worth noting that the ubiquitination sites on P53 are also targets of acetylation by HAT p300/CBP, and the association affinity of P53 and MDM2 is significantly affected by the acetylation [41].

NOC2L (amino acids 147-609) was found to bind directly to MDM2 in the central acidic and zinc finger domain (amino acids 222-366) and the N terminus (amino acids 1-122) of MDM2 [8]. Previous findings indicated that the association affinity between P53 and MDM2 is affected by acetylation [41]. NOC2L can block the acetylation of P53 [7] and MDM2 [8], as well as inhibit the ubiquitination of MDM2 [8]. We can speculate that NOC2L stabilizes the P53-MDM2 complex through this approach. However, the precise molecular basis of this process was not fully investigated in their study.

These above findings raised the possibility that NOC2L, P53 and MDM2 may constitute a ternary complex like the previous proposed P53-NOC2L-Aurora B model [7, 8, 42]. However, it was difficult to detect the ternary complex in vivo due to the small amount and the overlapping binding form of the complex [8].

As a result of the NOC2L association with MDM2, NOC2L facilitates MDM2-mediated P53 repression by sustaining the stability of MDM2 itself and the MDM2-P53 complex, which further represses the P53-mediated transcriptional activation significantly [8].

#### NOC2L regulates TAp63 in a cell cycle-controlled manner

As the investigation of NOC2L-mediated P53 regulation continued, scientists turned their attention to the role of NOC2L on other proteins homologous with P53 [11]. P63 resembles with P53 both genetically and functionally, as a member of the P53 family [43]. Under this note, further study was conducted to understand the association of NOC2L with P63 [11].

In addition to the P53-dependent tumor suppression function mentioned above, P63 shows significant ability to regulate epithelial development including cell growth and differentiation [44]. P63 constitutes multiple isoforms, including TA isoforms with a transactivation domain and ΔN isoforms without this domain [45]. In accordance with P53, P63-regualted transcriptional activity is associated with the participation of p300/CBP [46]. Evidence indicates that p300 can also acetylates TAp63, promoting the transcription of *P21* which is targeted by P63 [46].

NOC2L was verified to physically interact with TAp63 and affect its transcriptional activity in a negative way [11]. The binding region in NOC2L is its central portion while in TAp63 is the oligomerization domain [11]. Actinomcyin D (ActD) was previously reported to induce some nucleolar proteins to translocate to nucleoplasm [47]. When cells were treated with actinomcyin D which was previously reported to induce some nucleolar proteins to translocate to nucleoplasm[47], NOC2L was observed to translocate from the nucleus to the nucleoplasm and co-localized with TAp63 in the *P21* promoter region, resulting in the transcriptional inhibition of downstream genes in a P53 resembling way [11]. Throughout the cell cycle, the expression level of NOC2L peaks in G2/M phase and reaches the minimum in G1/S, suggesting a cell-cycle controlled characteristic of NOC2L [11].

In summary, NOC2L can regulate TAp63 activity negatively in a cell-cycle controlled manner [11]. Just as P53 can be considered as a toxin for a cell under physically conditions [7], the P53-relative TAp63 can also be regarded as a toxin whose toxic effect can be counteracted by the small fraction of NOC2L in the nucleoplasm [11].

#### NOC2L is involved in multiple biological events

Based on the knowledge of the biological properties and function of NOC2L, further investigation was conducted to examine how NOC2L affects biological events. It has been proposed that NOC2L can participate in some key biological events, including the rRNA biogenesis [48], embryonic development [49, 50], and cancer progression [9, 51]. All these biological events affected by NOC2L can be contributed to its characteristic and function mentioned previously in this review.

#### NOC2L participates in eukaryotic ribosome RNA processing

As mentioned previously, NOC2L predominantly exits in the nucleolus [7, 11]. However, the exact role of NOC2L plays in the nucleolus was still unclear. One important event happening in the nucleolus is the rRNA biogenesis [52]. During this process, multiple proteins and small RNAs are involved to direct the synthesis of the 40S and 60S subunits [52]. Among them, U3 snoRNA-associated proteins (UTPs) are responsible for the biogenesis of the 40S subunit [53]. Previous analysis of NOC2L-associated proteins showed that NOC2L interacts with a UTP named as Down Regulated in Metastasis (DRIM) [54], suggesting that NOC2L may participate in the biogenesis of rRNA.

Wu et. al confirmed that NOC2L is indeed involved in the processing of the 40S and 60S subunits of ribosome [48]. They first confirmed that NOC2L is primarily expressed and coexists with DRIM in the nucleolus [48], as indicated before. Upon further investigation, NOC2L was demonstrated to affect 18S, 28S and 5.8S rRNA processing because *NOC2L*-knockdown downregulated the levels of these rRNAs and other associated pre-rRNAs [48]. This observation further confirmed the participation of NOC2L in rRNA processing. Additionally, the rRNA processing happens at the same time with the assembly of pre-ribosomal particles. Based on this information, NOC2L was examined to coexist with the pre-40S and pre-60S ribosomal particles [48]. Meanwhile, 32S and 12S pre-rRNAs also associated with NOC2L in vivo [48], leading scientists to further investigate whether NOC2L also associates with U8 snoRNA which is a binding partner of 32S rRNA [52]. Upon further study, NOC2L was demonstrated to interact with U8 snoRNA but not change its level [48]. Since U8 snoRNA is known to be responsible for the 28S and 5.8S rRNA processing [55], NOC2L is considered to participate in 28S and 5.8S rRNA processing but does not affect U8 snoRNA level [48, 52, 55]. Similarly, NOC2L was later demonstrated to be involved in 18S rRNA processing and does not affect U3 snoRNA level [48].

As indicated before, NOC2L has been recognized as a transcriptional repressor [7–11]. Here researchers present another important role of NOC2L as a nucleolar protein in rRNA biogenesis [48]. However, the precise mechanisms of how NOC2L mediates rRNA processing remains to be further investigated in future studies.

#### NOC2L is associated with embryonic development

NOC2L has been recognized as a P53 suppressor [7], while P53 can be regarded as a toxin for a cell under normal conditions [56]. Take embryonic development for example, loss of P53 does not influence this process [57], while overexpression of p53 has a lethal effect on embryonic development [58]. NOC2L may be crucial for certain developmental events when P53 needs to be negatively regulated. Previous studies showed that NOC2L is expressed throughout mouse embryogenesis, and *NOC2L*-knockout mice all died during embryonic development [7]. Based on these findings, we may speculate that NOC2L is also related to the normal development of embryo.

#### NOC2L mediates early lymphocyte development

When cells undergo physiological DNA breaks during V(D)J recombination of lymphocytes, P53 is required to be suppressed temporarily in order to ensure the differentiation of T and B cells [59]. Since NOC2L has been recognized as a P53 suppressor, it is possible that NOC2L may contribute to the development of lymphocyte [50].

Mice bearing lymphoid-restricted deletion of *NOC2L* generated [50]. Results indicated that *NOC2L* deficient mice have a remarkable decrease in both double positive (DP) and single positive (SP) T lymphocytes while an increase in double negative (DN) T lymphocytes [50]. Scientists discovered a block between DN3 and DN4 stage, as well as a significant decrease in DN3L cells, which mainly stay in S and G2/M phase [50]. During the transition of DN3E to DN3L stage, cells undergo β-selection, and the failure of β-selection caused by NOC2L deletion led to the subsequent decrease in DN4, DP and SP T lymphocytes [50]. During T lymphocyte development, the physiological event of DNA breaks induces P53 to inhibit cell differentiation and development [60]. With the absence of NOC2L, P53 cannot be negatively regulated [7]. Upon *P53* knockdown, the defect described above partly but not fully recovered [50], suggesting NOC2L functions in a P53-dependent way in this event [7, 50].

In conclusion, this study indicates that NOC2L participates in early T-lymphocyte development by repressing P53 [50]. In addition to the early T lymphocyte developmental defects, early B-lymphocyte development is also blocked between the pro-B and pre-B stages [50]. All in all, NOC2L/P53 functions as a checkpoint cooperatively for the maturation of B and T lymphocytes [50, 56, 60].

#### NOC2L regulates epidermis development

Another important target of NOC2L is P63, as mentioned before [11]. As a close relative of P53, P63 shares common features and functions with P53, but what makes P63 unique is its ability to regulate epidermis development [61]. In this case, there is a possibility that NOC2L may also be involved in regulating epidermis development through the association with both P53 and P63 [7, 11, 49].

During the development of murine fetus, NOC2L is observed to express from embryonic day12.5 to postnatal day0 in all epidermal cells, suggesting its importance in epidermal development [49]. Following this observation, epidermis specific *NOC2L* conditional knockout (CKO) mice were generated for further investigation [49]. As a result, various severe epidermal defects were observed and the *NOC2L*^CKO^ mice died at birth [49]. The epidermis abnormalities observed include thin and smooth skin, absence of eyelid fusion, impairment of epidermis stratification, lack of hair follicle and detachment of epidermis during embryogenesis [49]. Further experimental results of epidermal stratification markers were in accordance with the previous discovery [49].

Taken together the above-mentioned defects observed in *NOC2L*^CKO^ mice, it is notable that they are similar to the phenotype of *P63* deficiency [44]. In this situation, it is possible that the epidermis abnormalities in *NOC2L*^CKO^ mice may be contributed to abnormal regulation of P63 [49]. Upon further investigation, P63 and its target gene *GATA3* were both demonstrated to be downregulated during embryogenesis [49]. Further studies indicated that NOC2L is recruited at the promoter region of P63 and inhibits the acetylation of H3K18 [49]. Since P63 was described to regulate proper orientation of cell division [62], downregulation of P63 caused by *NOC2L* deficiency leads to the incapacity to enter asymmetric cell division and complete stratification process [49].

Besides the investigation of how NOC2L regulates epidermis development through P63, the role of P53 was also considered. As mentioned before, NOC2L can inhibit the acetylation of P53 [7], therefore suppressing its function. During the investigation of the epidermis development of *NOC2L*^CKO^ mice, it was further confirmed that the abnormalities in epidermis development were partially contributed to P53 hyper-acetylation [49]. Since both P53 and P63 are involved in cell proliferation and apoptosis [63], the *NOC2L*-deficient epidermal cells experienced impaired cell proliferation and enhanced apoptosis [49].

In conclusion, NOC2L plays an essential role in regulating epidermis development by association with both P53 and P63.

#### NOC2L promotes cancer progression

As a negative regulator of tumor suppressor P53 [7], NOC2L may promote tumorigenesis by counteracting the tumor suppression function of P53 [7]. In addition to P53-dependent pro-cancer effect of NOC2L [9, 51], other P53-independent mechanisms of NOC2L-mediated tumorigenesis remain to be further addressed. As we all know, uncontrolled proliferation is a typical characteristic of malignant tumor [64]. *NOC2L* knock-out has been found to significantly repress cell proliferation in the goal of the Dependency Map (DepMap) database [65], indicating that NOC2L is a potential oncogene.

**Fig. 6:**
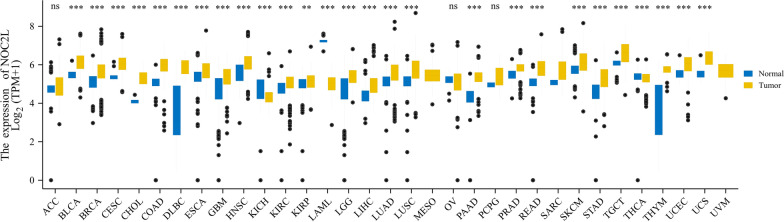
The Comparison of *NOC2L* Expression Between Tumor and Normal Samples. (The data source of this statistical analysis is the UCSC Xena project (http://xena.ucsc.edu/), which integrated data from TCGA and GTEx database. Mann–Whitney U test (Wilcoxon rank sum test) was used to compare the expression of NOC2L between normal and tumor samples (ns, p ≥ 0.05; *,p < 0.05; **,p < 0.01;***,p < 0.001). *ACC* Adrenocortical carcinoma, *BLCA* Bladder Urothelial Carcinoma, *BRCA* Breast invasive carcinoma, *CESC* Cervical squamous cell carcinoma and endocervical adenocarcinoma, *CHOL* Cholangiocarcinoma, *COAD* Colon adenocarcinoma, *DLBC* Lymphoid Neoplasm Diffuse Large B-cell Lymphoma, *ESCA* Esophageal carcinoma, *GBM* Glioblastoma multiforme, *HNSC* Head and Neck squamous cell carcinoma, *KICH* Kidney Chromophobe, *KIRC* Kidney renal clear cell carcinoma, *KIRP* Kidney renal papillary cell carcinoma, *LAML* Acute Myeloid Leukemia, *LGG* Brain Lower Grade Glioma, *LIHC* Liver hepatocellular carcinoma, *LUAD* Lung adenocarcinoma, *LUSC* Lung squamous cell carcinoma, *MESO* Mesothelioma, *OV* Ovarian serous cystadenocarcinoma, *PAAD* Pancreatic adenocarcinoma, *PCPG* Pheochromocytoma and Paraganglioma, *PRAD* Prostate adenocarcinoma, *READ* Rectum adenocarcinoma, *SARC* Sarcoma, *SKCM* Skin Cutaneous Melanoma, *STAD* Stomach adenocarcinoma, *TGCT* Testicular Germ Cell Tumors, *THCA* Thyroid carcinoma, *THYM* Thymoma, *UCEC* Uterine Corpus Endometrial Carcinoma, *UCS* Uterine Carcinosarcoma, *UVM* Uveal Melanoma)

The Cancer Genome Atlas (TCGA) database was exploited to analyze the expression of *NOC2L* between cancer and cancer adjacent tissues [66]. We found that *NOC2L* increased in most cancer tissues than their counterpart, suggesting that it may be an oncogene (Fig. 6; Additional File 1). Further survival analysis indicated that the difference in OS (Overall Survival) between NOC2L-high and NOC2L-low patients is statistically significant in several types of cancers, including adrenocortical carcinoma, bladder urothelial carcinoma, head and neck squamous cell carcinoma, brain lower grade glioma, liver hepatocellular carcinoma and sarcoma(p < 0.05). An additional file shows the detailed information of the survival analysis [see Additional file 2].

#### NOC2L promotes colorectal cancer via association with RB

TCGA database and human colorectal cancer (CRC) tissues analysis both indicate that NOC2L is upregulated in CRC. Furthermore, the level of NOC2L is significantly related to the poor outcome of CRC patients. These evidences imply that NOC2L may play an important role in the tumorigenesis and progression of CRC [9].

Retinoblastoma protein (RB) is another key tumor suppressor and its downregulation or inactivation have been demonstrated to facilitate CRC [67–69]. NOC2L has been identified as an RB binding partner by both bioinformatics analysis and in vivo experiments [9], and the binding motif is the LxCxE motif in its INHAT2 domain [9]. NOC2L has been demonstrated to downregulate RB level by directly interacting with RB instead of regulating its transcription [9]. Upon further investigation, it was described that NOC2L promotes RB degradation and ubiquitination probably via repressing RB acetylation [9]. However, further investigation is needed to verify this statement and identify the acetylation site inhibited by NOC2L in RB.

In both cell and mouse xenograft, NOC2L has been demonstrated to promote tumor growth and cancer cell proliferation through RB [9], confirming its role in tumorigenesis of CRC. In conclusion, NOC2L might be a potential marker, predictor, and therapeutic target for CRC in future clinical practice [9].

#### NOC2L promotes breast cancer via regulating FOXO3

As a common cancer among females, breast cancer is one of the most studied cancers [70]. Multiple tumor promotion and suppression genes have been investigated to further the understanding of the precise molecular mechanism of breast cancer [71].

NOC2L was demonstrated to be upregulated in breast cancer patients [51]. Moreover, the high expression level of NOC2L was correlated with the poor outcome of breast cancer patients [51]. In breast cancer cells, cell proliferation and colony formation was suppressed upon *NOC2L* knockdown, indicating an important role of NOC2L in breast cancer progression [51].

NOC2L was found to be recruited by Enhancer of zeste homolog 2 (EZH2) and cooperate with EZH2 through physical interaction to promote the methylation of H3K27 in the vicinity of forkhead box O3 (*FOXO3*) promoter region [51], leading to the downregulation of tumor suppressor FOXO3 in breast cancer cells [51]. As a consequence of FOXO3 downregulation, tumor progression was promoted [51]. Similar to the situation of CRC [9], NOC2L may also be a potential marker and target for breast cancer prediction and treatment [51].

## Conclusion and future perspectives

The investigation of NOC2L provides a novel perspective on the histone acetylation mechanisms, as is summarized in Table 1. However, studies on NOC2L are insufficient in number and some of them are relatively superficial. The molecular mechanism of NOC2L inhibition of HAT is not studied deeply enough, and the relationship between NOC2L and HAT should be explored in depth. There is possibility that NOC2L influences the expression or activity of HAT. NOC2L may directly target a certain structure of HAT, or indirectly target HAT through other proteins to inhibit HAT activity. Additionally, kinetic experiments may be conducted to compare the binding affinity between NOC2L and HAT. In this review, several studies only demonstrated the effects of NOC2L on cellular events but failed to elaborate the relationship between its basic INHAT function and these effects. Therefore, deeper investigation is highly required to understand the biological function of this gene and enhance the knowledge of histone acetylation regulation. Since literature reporting NOC2L is still limited, we turned to online database for more information. Using Bioplex3.0 database, we discovered 118 NOC2L-associated proteins in total and several NOC2L-related pathways [72], which may shed light on our future investigation.

**Table 1:** Summary of NOC2L roles in biological events

NOC2L-associated molecules	Direct effect by NOC2L	Biological events
P53	P53 acetylation↓ ** → **P53 activity↓	Apoptosis inhibition Cell-cycle arrest inhibition
MDM2	P53 ubiquitination↑ → P53 activity↓	
Aurora B	P53 phosphorylation↑ ** →**P53 activity↓	
P63	P63-occupied histone acetylation↓ ** → **P63-denpendent transcription↓	Apoptosis inhibition Cell-cycle arrest inhibition Cell division dysfunction
RB	RB acetylation↓ ** → **RB activity↓	Breast cancer proliferation
EZH2	EZH2-occupied histone acetylation↓ ** → **FOXO3 transcription↓	Colorectal cancer proliferation

As we dig deeper into the regulation mechanism of NOC2L, more questions and hypothesis are raised. Previous studies all focused on the downstream targets of NOC2L. Therefore, there is lack of information on the upstream regulatory molecules of NOC2L, which gives us a direction for future investigation. For example, microRNA, long-noncoding RNA and other transcriptional factors targeting *NOC2L* promotor all have the potential to regulate NOC2L expression.

In addition to the molecular mechanism of NOC2L regulation, technical means are required to innovate so that we can generate globally *NOC2L*-knockout mice for further investigation. All these issues require further investigation.

According to the biological function of NOC2L, there is hope that NOC2L may be explored as a potential biomarker of cancer and more importantly a therapeutic target in clinical practice. In conclusion, the precise mechanisms of how NOC2L functions remain to be elucidated in future studies, and the investigation of other important NOC2L-associated proteins is still needed.

## Supplementary Information

**Additional file 1**: NOC2L expression in each sample. This file contains the expression of NOC2L in each tumor and normal sample. The data source of these samples is the UCSC Xena project which integrated data from TCGA and GTEx database.

**Additional file 2**: Overall survival analysis of NOC2L across pan-cancer. This file shows that the difference in OS (Overall Survival) between NOC2L-high and NOC2L-low patients is statistically significant in several types of cancers, including adrenocortical carcinoma, bladder Urothelial Carcinoma, head and neck squamous cell carcinoma, brain Lower Grade Glioma, liver hepatocellular carcinoma and sarcoma(p<0.05). Other types of cancers show no significant differences of OS.

## Data Availability

The datasets generated during and/or analyzed during the current study are available in the TCGA database, https://www.cancer.gov/tcga [66].

## Abbreviations

NOC2L: NOC2 like nucleolar associated transcriptional repressor
INHAT: Inhibitor of histone acetyltransferase
HAT: Histone acetyltransferase
HDAC: Histone deacetylases
BLAST: The basic local alignment search tool
NoLS: Nucleolar localization sequence
NoD: Nucleolar localization sequence detector
DBD: DNA-binding domain
DOX: Doxorubicin
MDM2: Mouse double minute 2 homolog
ActD: Actinomcyin D
UTPs: U3 snoRNA-associated proteins
DRIM: Down regulated in metastasis
CKO: Conditional knockout
DP: Double positive
SP: Single positive
DN: Double negative
TCGA: The cancer genome atlas
ACC: Adrenocortical carcinoma
BLCA: Bladder urothelial carcinoma
BRCA: Breast invasive carcinoma
CESC: Cervical squamous cell carcinoma and endocervical adenocarcinoma
CHOL: Cholangiocarcinoma
COAD: Colon adenocarcinoma
DLBC: Lymphoid neoplasm diffuse large B-cell lymphoma
ESCA: Esophageal carcinoma
GBM: Glioblastoma multiforme
HNSC: Head and Neck squamous cell carcinoma
KICH: Kidney chromophobe
KIRC: Kidney renal clear cell carcinoma
KIRP: Kidney renal papillary cell carcinoma
LAML: Acute myeloid leukemia
LGG: Brain lower grade glioma
LIHC: Liver hepatocellular carcinoma
LUAD: Lung adenocarcinoma
LUSC: Lung squamous cell carcinoma
MESO: Mesothelioma
OV: Ovarian serous cystadenocarcinoma
PAAD: Pancreatic adenocarcinoma
PCPG: Pheochromocytoma and paraganglioma
PRAD: Prostate adenocarcinoma
READ: Rectum adenocarcinoma
SARC: Sarcoma
SKCM: Skin cutaneous melanoma
STAD: Stomach adenocarcinoma
TGCT: Testicular germ cell tumors
THCA: Thyroid carcinoma
THYM: Thymoma
UCEC: Uterine corpus endometrial carcinoma
UCS: Uterine carcinosarcoma
UVM: Uveal melanoma
RB: Retinoblastoma protein
CRC: Colorectal cancer
EZH2: Enhancer of zeste homolog 2
FOXO3: Forkhead box O3
AURKB: Aurora kinase B
HPA: Human protein atlas
DEPMAP: The goal of the dependency map
GTEx: Genotype-tissue expression
