# Supplementary figures and images for: Unmasking the biological function and regulatory mechanism of NOC2L: a novel inhibitor of histone acetyltransferase
Source: J Transl Med. 2023 Jan 17;21:31. doi: 10.1186/s12967-023-03877-2 (PMC9844006; doi:10.1186/s12967-023-03877-2)

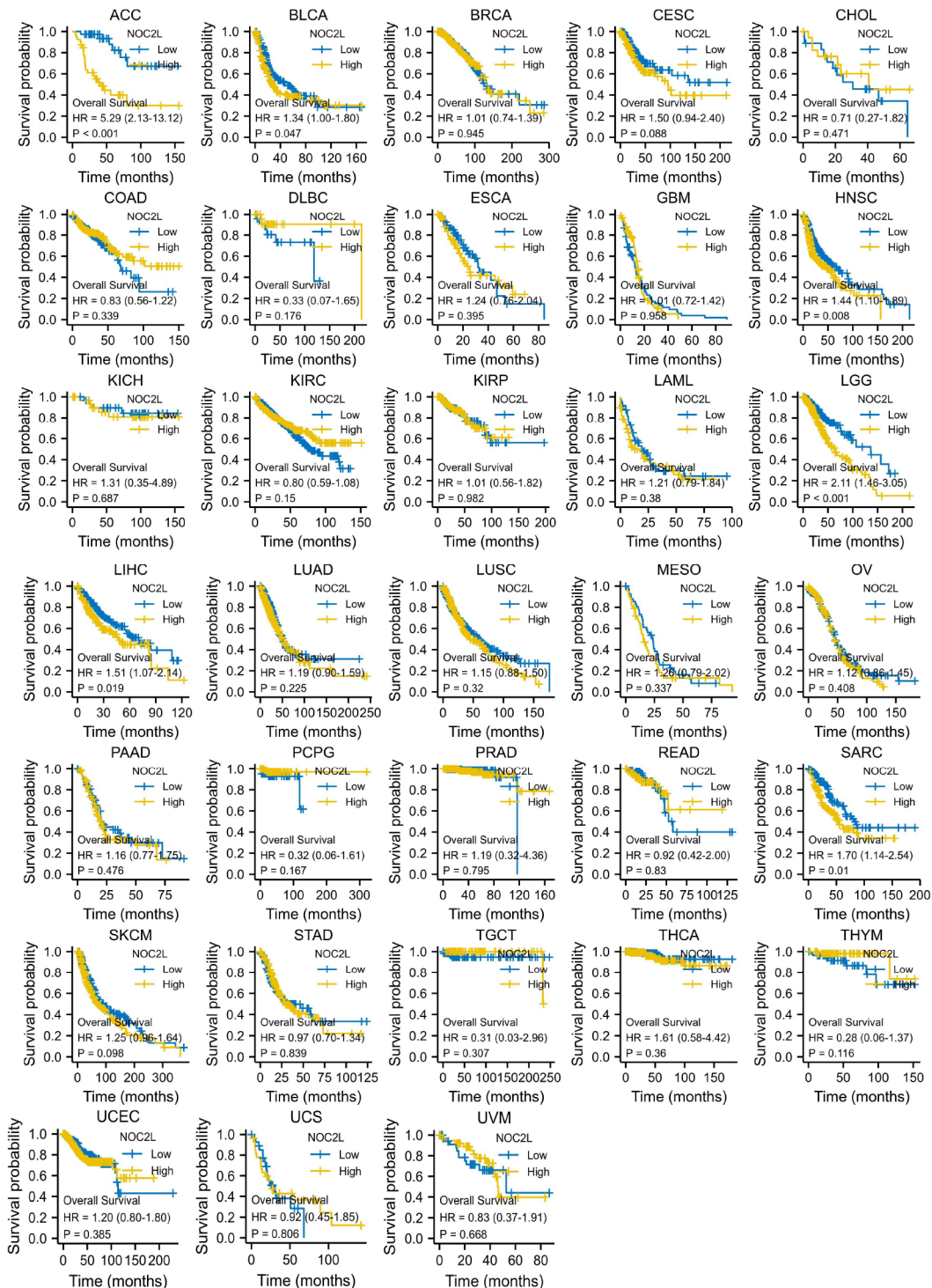

Supplement: Supplementary file 2 — Additional file 2: Overall survival analysis of NOC2L across pan-cancer. This file shows that the difference in OS (Overall Survival) between NOC2L-high and NOC2L-low patients is statistically significant in several types of cancers, including adrenocortical carcinoma, bladder Urothelial Carcinoma, head and neck squamous cell carcinoma, brain Lower Grade Glioma, liver hepatocellular carcinoma and sarcoma(p<0.05). Other types of cancers show no significant differences of OS. [file 12967_2023_3877_MOESM2_ESM.pdf]
